# Surface Transition on Ice Induced by the Formation of a Grain Boundary

**DOI:** 10.1371/journal.pone.0024373

**Published:** 2011-09-07

**Authors:** Christian Pedersen, Albert Mihranyan, Maria Strømme

**Affiliations:** Department of Engineering Sciences, Uppsala University, Uppsala, Sweden; Massey University, New Zealand

## Abstract

Interfaces between individual ice crystals, usually referred to as grain boundaries, play an important part in many processes in nature. Grain boundary properties are, for example, governing the sintering processes in snow and ice which transform a snowpack into a glacier. In the case of snow sintering, it has been assumed that there are no variations in surface roughness and surface melting, when considering the ice-air interface of an individual crystal. In contrast to that assumption, the present work suggests that there is an increased probability of molecular surface disorder in the vicinity of a grain boundary. The conclusion is based on the first detailed visualization of the formation of an ice grain boundary. The visualization is enabled by studying ice crystals growing into contact, at temperatures between −20°C and −15°C and pressures of 1–2 Torr, using Environmental Scanning Electron Microscopy. It is observed that the formation of a grain boundary induces a surface transition on the facets in contact. The transition does not propagate across facet edges. The surface transition is interpreted as the spreading of crystal dislocations away from the grain boundary. The observation constitutes a qualitatively new finding, and can potentially increase the understanding of specific processes in nature where ice grain boundaries are involved.

## Introduction

The importance of water on earth, and in the atmosphere, has made it one of the most investigated molecules. Ice crystals are involved in the production of thunderstorms [1], affect the destruction of ozone in the stratosphere [2] and constitute the snow that falls to the ground. Interfaces between individual ice crystals are referred to as grain boundaries, and grain boundaries are involved in many processes in nature [3] (throughout this paper, the term *grain boundary* does not include the ice-air interface). The properties of ice grain boundaries are unknown to a large extent, much due to the difficulty in investigating a grain boundary at thermodynamic equilibrium [3], [4]. It is for example uncertain to what extent grain boundaries affect the structure of ice surfaces in their vicinity [5].

The thermodynamically stable form of ice at ambient temperature and pressure is ice Ih, which has a hexagonal crystal structure [6]. Hexagonal prism growth is the basic growth morphology of ice Ih, and the growth of simple ice prisms has therefore undergone much investigation [7]. Ice crystals growing into contact has been investigated previously [8]–[10], but the process of two ice prisms growing into contact with each other has not been visualized in detail prior to this work (i.e. growth from vapor). Such an experiment would provide basic information on how facets are affected by an adjacent grain boundary.

## Results

This paper explores the possibility of visualizing two ice prisms growing into contact, using Environmental Scanning Electron Microscopy (ESEM). ESEM is a technique that operates at higher pressure than does regular SEM, and its principal of detection takes advantage of a signal amplification from secondary electrons produced by electron-gas interactions [11]. The gas used in the present work was pure water vapor. Ice crystallisation was controlled by careful adjustments of chamber pressure and sample stage temperature. The experiments were conducted by initially lowering pressure and temperature to a condition at which ice sublimation occurs (0.2–0.5 Torr, and between −15°C and −20°C) to remove ice from the sample, and subsequently raising the pressure stepwise until crystal growth was observed. Ice crystal growth was studied on two types of surfaces: the stainless steel surface of an ESEM sample stage, and the surface of a polyvinyl alcohol cryogel.

**Figure 1:**
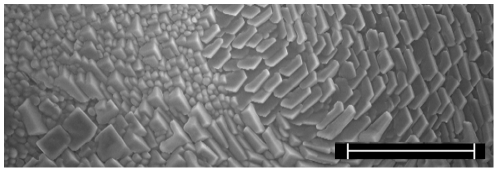
Ice crystals formed on the stainless steel surface of an ESEM sample stage. The water vapor density is 1.5 Torr, and the temperature of the sample stage is −15°C. The scale bar is 100 µm.

On the stainless steel surface, the average distance between nucleation sites was relatively short, which prohibited studying the growth of individual crystals; every crystal was in contact with another crystal already as the first image was recorded, as shown in Figure 1. The observation of relatively short distance between nucleation sites is in analogy with previous ESEM experiments, where the formation of ice was investigated on other solid surfaces [2], [12].

**Figure 2:**
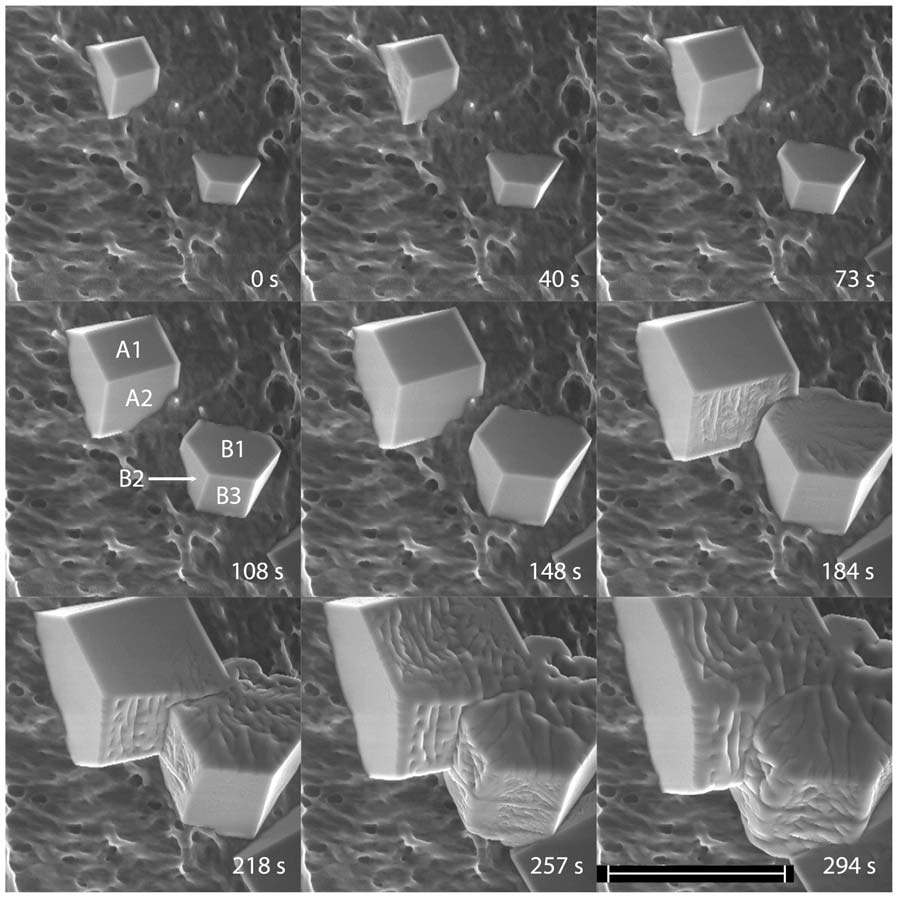
Crystals of hexagonal ice (ice Ih) growing on the surface of a polyvinyl alcohol cryogel. The Figure shows that facets which come into contact with another crystal undergo a surface transition. For clarity, facets of interest are named in the 108 s picture. At 184 s, facets A2 and B1 has grown into contact and undergone a surface transition. At 218 s, facet B2 has undergone a surface transition. At 257 s, facets A1 and B3 have grown into contact with other crystals and undergone surface transitions (facet B3 is in contact with the crystal in the lower right corner). The chamber pressure of water vapor is 1.2 Torr. The temperature of the sample stage is −20°C, and the crystal surface temperature is estimated to between −16.2°C and −17.4°C (see Methods section). The scale bar is 100 µm. An extended version of this image series is given in the *Supporting Information S1*.

When ice crystal growth was investigated on the surface of a polyvinyl alcohol cryogel, nucleation occurred at much fewer sites as compared to the stainless steel surface, which enabled monitoring the growth of individual ice crystals. The crystals generally grew with hexagonal prism morphology, with sharp facet edges and smooth facets, until they came into contact with another crystal. It was observed that the transition of a facet structure from smooth to wavy could be catalysed by a contact with an adjacent crystal, as shown in Figure 2. An extended version and discussion of Figure 2 is presented in *Supporting Information S1*. The facet transition exemplified in Figure 2 was only observed on facets directly after they had come into contact with another growing crystal (see also Figure S5 and discussion in *Supporting Information S1*). For the largest facets observed (facet edges within the range 200–300 µm), the transition from smooth to wavy of the entire facet occurred faster than the time interval between image acquisitions (i.e. 30–40 s), as shown in Figure S3. The transition did not propagate across facet edges to adjacent facets, as shown in Figure 2. Furthermore, it was observed that the transition increased the crystal growth rate, as shown in Figure S4.

## Discussion

The results clearly show that it was the *contact* between crystals and not, e.g., a certain temperature which initiated the transition, since the transition only was observed on facets directly after they had come into contact with another crystal (see observations made at different temperatures; Figure S1 and Figure S2). In addition, it is unlikely that the observed transition should be induced by heat flow between the two crystals in contact (i.e. slightly different surface temperatures before contact); the transition always halted at a facet edge, whereas heat is expected to flow through the crystal in three dimensions and not halt at facet edges. It could also be mentioned, in this context, that the observed transitions can not be the result of a pressure forcing the crystals together, since the crystals investigated in this work have grown into contact (contact load is known as a factor that accelerates ice sintering [13], [14]).

Images of ice crystals growing into contact has been presented prior to this work, in investigations of epitaxial ice crystal growth on covellite substrates, studied with light microscopy [8], [10]. In those studies, ice crystals were growing on a single-crystalline substrate with their basal facets parallel to the substrate. The studies showed that contact between two ice crystals facilitates incorporation of molecules into the crystal structure, by the formation of new molecular layers at the grain boundary which spread laterally away from the grain boundary. Consequently, the contact was observed to accelerate growth and transform smooth basal facets into slopes or stair-case like structures (so-called Hopper development [10], [15]). The present results, however, clearly show that contact between crystals transforms facets into wavy structures (i.e. not slopes or staircase-like structures), which means that new molecular layers *are not* predominantly initiated at the grain boundary (see Figure 2). The present work differs from the previous studies in a number of ways. The present work is an electron microscopy study performed at low pressure, and the magnification and resolution are considerably higher as compared to the previous studies performed with light microscopy. Furthermore, in the present study, the crystal lattices of two crystals in contact are randomly oriented relative to each other (the basal facets were parallel to each other in the previous studies). When two randomly oriented crystals are in contact, the grain boundary can be viewed as an array of dislocations [16], and it is known that the presence of one dislocation facilitates the formation of additional dislocations [17]. The surface transition observed in the present study is therefore interpreted as the spreading of crystal dislocations away from a grain boundary during ice growth.

It is difficult to comment on the dislocation density of the transforming facets. One possibility is that the “distances between the waves” on the wavy surface structure are connected to the distances between dislocation layers. Another possibility is that the dislocation density is very high, and that the surface close to the boundary can be compared to a melted condition. The theory of *dislocation-mediated melting* has indeed suggested that continuous melting transitions can occur as “an avalanche of dislocations”, propagating away from the original dislocation [17]. Such an “avalanche” is brought about by a lowering of the energy of creation of additional dislocations, in the presence of the original dislocation. Grain boundaries are arrays of crystal dislocations, and they can in this work be viewed as the original dislocations.

To the best of our knowledge, no theoretical model has been presented which describe the spreading of crystal dislocations away from a grain boundary. It is also beyond the scope of the present work to create such a model; the purpose of this report is the rapid communication of a qualitatively new finding. The finding will potentially improve the understanding of processes in nature where ice grain boundaries are involved; one example is the sintering of snow. In the case of snow sintering, it is unknown whether or not the surface structure varies over the ice-air surface of an individual grain [5]. Today's theoretical snow sintering models assume that there are no variations in surface roughness and surface melting, when considering the surface of a single grain [5]. However, the results presented here show an example of ice crystals in contact where the ice-air surface structure varies strongly over each individual grain; a grain boundary-induced surface transition is observed, which does not propagate across facet edges. The results therefore suggest that it is *possible* that the probability of molecular surface disorder is increased in the vicinity of a grain boundary. At present, we do not know how generalizable the results are, e.g. if similar grain boundary-induced transitions can occur at conditions found in nature. We do not know how far the surface transition would spread if the crystal surface was slightly curved before transition (instead of a smooth facet), and we do not know if similar transitions can occur at ambient pressure. Unfortunately, those conditions can not be investigated with the present experimental setup. Nevertheless, the observed transitions are caused by the *contact* between two crystals, i.e. two crystal lattices which “mismatch” at the grain boundary, and the mismatch is the same regardless of surrounding pressure. It should also be kept in mind that prism growth is the basic growth morphology of ice Ih, and all understanding of more complex growth morphologies (e.g. the crystal morphologies found in a snowpack) is based on the understanding of simple prism growth. To summarize the interpretation of the results, they suggest the possibility that there is an increased probability of molecular surface disorder in the vicinity of a grain boundary.

When exemplifying the impact the observations might have on the understanding of phenomena in nature, it should be recalled that the grain boundary-induced transition is connected to an increased crystal growth rate (Figure S4). One of the major mass transfer mechanisms in snow sintering is vapor transport [5], [18]–[20], and vapor transport can - a bit simplified - be summarized in three steps: (i) evaporation at the ice surface “far away” from the grain boundary, (ii) water vapor diffusion towards the grain boundary and (iii) condensation of water vapor close to the grain boundary. It has been argued that the latest sintering models underestimate the influence of vapor transport [5], and it is therefore possible that the present findings implicate that the models can be improved; an increased probability of molecular surface disorder close to a grain boundary would facilitate water attachment in the vicinity of the grain boundary, and thereby accelerate the vapor transport [7]. In particular, the present results could be valuable for the understanding of the early stages of “dry snow sintering”, since those stages show the strongest resemblance to the presently investigated system (e.g. temperature, grain morphology).

In summary, this work shows that the formation of an ice grain boundary can be visualized in detail using ESEM. Experiments are shown where the formation of a grain boundary induces a surface transition on facets which grow into contact, and the transition does not propagate across facet edges. The surface transition is interpreted as the spreading of crystal dislocations away from the grain boundary. The fact that an ice grain boundary can induce a surface transition which does not propagate across facet edges constitutes a novel qualitative finding. The results suggest the possibility that there is an increased probability of molecular surface disorder in the vicinity of a grain boundary. Previous studies of ice grain boundaries have mostly been attempts to investigate grain boundaries at thermodynamic equilibrium; this work shows that information on ice grain boundary properties also can be gained by studying the formation of a grain boundary during ice growth.

## Methods

Cryogels of polyvinyl alcohol, PVA, were produced by dissolving 6 wt% PVA (Mw 89,000–98,000) in water, and the solution was subjected to 7 freeze-thaw cycles (each cycle contained approximately 20 hours at −20°C followed by 4 hours at room temperature). Images of ice crystals were recorded using a Philips ESEM-FEG XL30, operating in wet mode. The environmental gas used was pure water vapor. PVA cryogels, 1–2 mm thick and containing water, were placed on a sample stage (a Peltier cooler stage with a stainless steel surface), and the pressure was first lowered and then raised to replace air with water vapor. The pressure was thereafter lowered to 0.2–0.5 Torr while simultaneously lowering the temperature of the sample stage to between −15°C and −20°C. The lowering of temperature and pressure caused ablation of water from the surface layer of the cryogel. The pressure was subsequently re-raised stepwise by letting in water vapor, 0.1 Torr/step, until the growth of ice crystals was observed. Ice crystal growth was studied within the pressure range 0.9–1.6 Torr, and images of individual ice crystals could be recorded with roughly 30 s intervals. The process of two crystals growing into contact with each other was investigated more than 100 times, at sample stage temperatures between −15°C and −20°C.

After investigating crystal growth, the pressure was lowered stepwise, 0.1 Torr/step, in order to study at which pressure crystal growth converted to ablation. The result was then used to estimate the crystal surface temperature [21], [22]. For the images exhibited in Figures 2 and S1, which were recorded with a sample stage temperature of −20°C, the crystal surface temperature is estimated to between −16.2°C and −17.4°C, based on the fact that crystal growth converted to ablation when the pressure was decreased from 1.1 to 1.0 Torr (Figure S1).

## Supporting Information

Supporting Information S1 **Supporting material text.**
(DOC)

Figure S1 **Crystals of hexagonal ice (ice Ih) growing on the surface of a polyvinyl alcohol cryogel.** The temperature of the sample stage is −20°C and the pressure of the sample chamber is given in the pictures. Crystal growth converts to ablation as the pressure is lowered from 1.1 to 1.0 Torr, and the temperature of the crystal surface can therefore be estimated to between −16.2°C and −17.4°C. The scale bar is 50 µm.
(TIF)

Figure S2 **An example of grain boundary-induced surface transition, observed at a sample stage temperature of −15°C.** The crystal surface temperature is estimated to between −13.6°C and −14.5°C. The water vapor density is 1.6 Torr. The scale bar in (d) is 100 µm.
(TIF)

Figure S3 **An example of a facet which grew to have edges longer than 200 µm before it came into contact with another crystal.** In Figure S3b the facet appears perfectly smooth. In Figure S3c, the upper part of the facet has come into contact with another crystal, and the whole facet has undergone a surface transition. The sample stage temperature was −18.5°C, and the temperature of the crystal surface is estimated to between −17.3°C and −18.5°C.
(TIF)

Figure S4 **Increase in the linear growth rate of the facets which undergo grain boundary-induced surface transition.** The figure shows two crystals that grow into contact, referred to as *the left* and *the right* crystal. For the left crystal, facets are named F1 and F2 while facet edges are named x and y, as shown in (a). Contact between the crystals is first observed in (l), and the contact results in a surface transition of facet F2. The x/y ratio of facet F1 is constant before the two crystals come into contact, but changes after contact is reached. The change in x/y ratio can be attributed to an increasing linear growth rate of facet F2. The water vapor density was 0.9 Torr. The temperature of the sample stage was −20°C, and the temperature of the crystal surface is estimated to between −18.4°C and −19.7°C. The scale bar in (p) is 50 µm.
(TIF)

Figure S5 **An example of a facet which develops an irregularity without contact with another crystal.** Such irregularities generally disappeared relatively fast, as exemplified in this figure: the right facet appears perfectly smooth in (a–b), exhibits an irregularity in (c–d) and appears perfectly smooth in (e–f). The water vapor density was 0.9 Torr. The temperature of the sample stage was −20°C, and the temperature of the crystal surface is estimated to between −18.4°C and −19.7°C. The scale bar in (f) is 50 µm.
(TIF)
