# Correction: Surface Transition on Ice Induced by the Formation of a Grain Boundary

**DOI:** 10.1371/annotation/51bc9350-b475-4b46-88b4-8bc1586d9d42

**Published:** 2013-10-25

**Authors:** Christian Pedersen, Albert Mihranyan, Maria Strømme

Supporting Information S1 was incorrectly attached. Please view the correct file for S1 here: 
