# Supplementary material for: Surface Transition on Ice Induced by the Formation of a Grain Boundary
Source: PLoS One. 2011 Sep 7;6(9):e24373. doi: 10.1371/journal.pone.0024373 (PMC3168470; doi:10.1371/journal.pone.0024373)
Supplement: Supporting Information S1 — Supporting material text. (DOC) [file pone.0024373.s001.doc]

**Supporting material**

The surface temperature of the ice crystals investigated in this work is higher than the sample stage temperature, during crystal growth: (i) the cryogel substrate constitutes a distance between the crystal and the cooling sample stage, (ii) crystallisation is exothermic, and (iii) the electron beam transfers some heat to the sample. The surface temperatures of the investigated facets were estimated by decreasing the pressure stepwise, 0.1 Torr/step, and observing at which pressure crystal growth converted to ablation. An example of this is given in Figure S1 (which is an extended version of Figure 2). In Figure S1 the sample stage temperature is –20ºC, and the crystals initially grow at a pressure of 1.2 Torr. After 294 s the pressure was lowered to 1.1 Torr, and crystal growth proceeded also at that pressure. After 451 s the pressure was lowered to 1.0 Torr, which changed the ongoing process from crystal growth to ablation. This change is essentially a change from a supersaturated condition to a subsaturated condition [22]. The result was used to estimate the crystal surface temperature during growth [21].

Figures 2 and S1 show an example of grain boundary-induced surface transition, observed at an estimated facet temperature between –16.2ºC and –17.4ºC, and a sample stage temperature of –20ºC. Figure S2 shows the same phenomenon at an estimated facet temperature between

–13.6ºC and –14.5ºC, and a sample stage temperature of –15ºC. The fact that the surface transition occurs at different temperatures shows that the transition is not induced by a specific temperature. Furthermore, the transition can not be induced by a heat flow from one crystal to the other (i.e. slightly different temperatures between the crystals); if the transition was induced by a heat flow, it would be expected to propagate across facet edges.

At rare occasions, a facet grew to have edges longer than 200 µm before it came into contact with another crystal. It was observed for such large facets, in analogy with observations of smaller facets, that contact with another crystal induced a transition of the whole facet, as exemplified in Figure S3. In Figure S3b the facet appears perfectly smooth. In Figure S3c, the upper part of the facet has come into contact with another crystal, and the entire facet has undergone a surface transition.

Figure S4 shows that a facet transition, caused by contact with another crystal, increases the linear growth rate of the facet (linear growth is growth in the direction perpendicular to a smooth facet). In Figure S4, facet F2 undergoes a surface transition. It is clear that the transition of F2 increases the linear growth rate of F2, since the proportions of F1 (the x/y ratio) changes as a response to the transition. This increase in linear growth rate was the general observation throughout the work.

Occasionally, irregularities developed on facets without contact with other crystals. These irregularities generally disappeared relatively fast, as shown in Figure S5. A more dramatic example of a crystal irregularity that disappeared fast is shown in Figure S4i-j (the upper right crystal). The appearance of such irregularities should be connected to the interaction between ice and cryogel. One important difference should be pointed out, between the irregularities exemplified in Figure S5 and the grain boundary-induced surface transitions shown in the other figures: the grain boundary-induced transition did generally not revert to smooth facets. However, at rare occasions, a facet was observed to remain nearly perfectly smooth, despite growing into contact with another crystal; an example of such an exception is shown in Figure S4k-p (the basal facet of the upper right crystal).
