# Supplementary figures and images for: Surface Transition on Ice Induced by the Formation of a Grain Boundary
Source: PLoS One. 2011 Sep 7;6(9):e24373. doi: 10.1371/journal.pone.0024373 (PMC3168470; doi:10.1371/journal.pone.0024373)

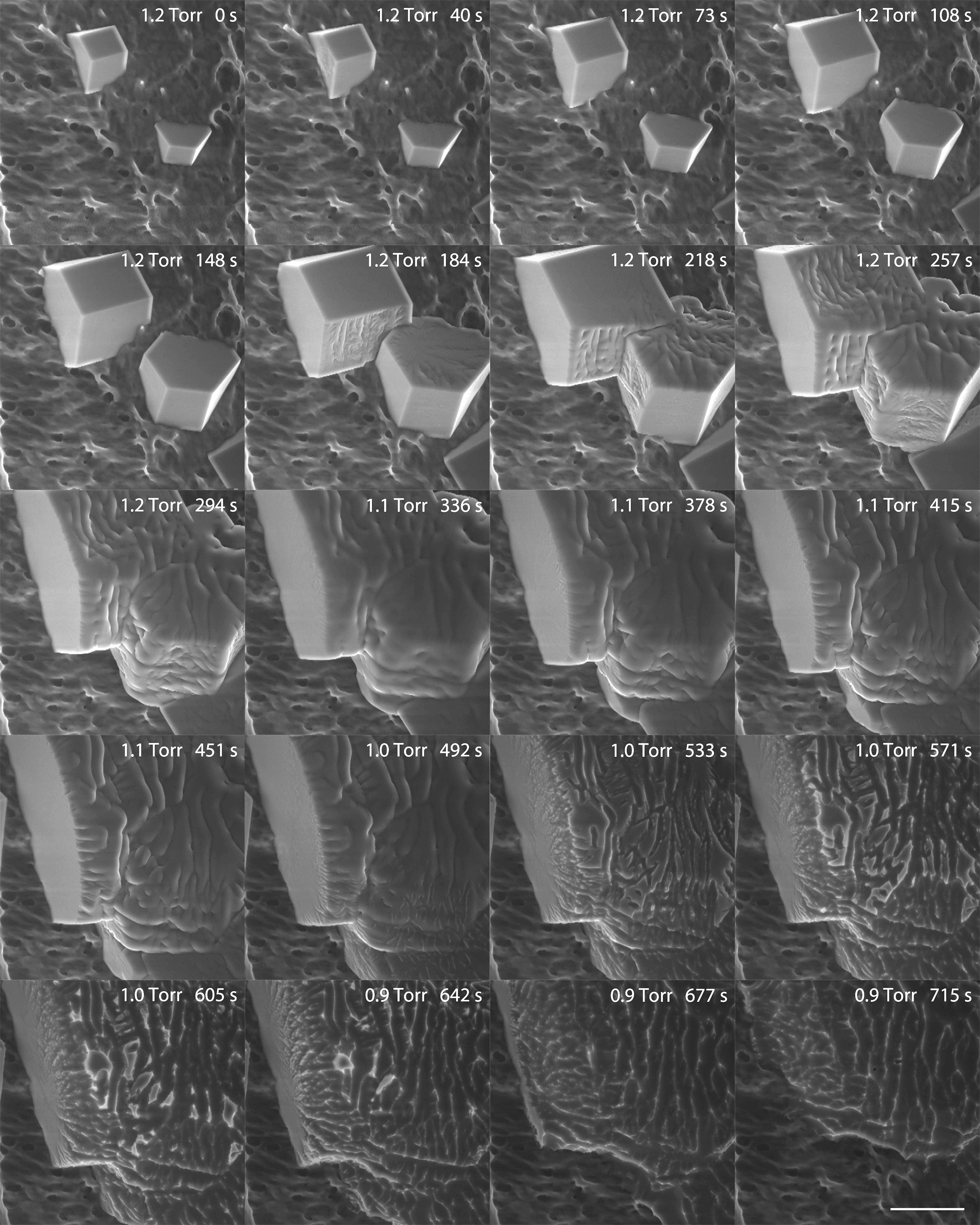

Supplement: Figure S1 — Crystals of hexagonal ice (ice Ih) growing on the surface of a polyvinyl alcohol cryogel. The temperature of the sample stage is −20°C and the pressure of the sample chamber is given in the pictures. Crystal growth converts to ablation as the pressure is lowered from 1.1 to 1.0 Torr, and the temperature of the crystal surface can therefore be estimated to between −16.2°C and −17.4°C. The scale bar is 50 µm. (TIF) [file pone.0024373.s002.tif]

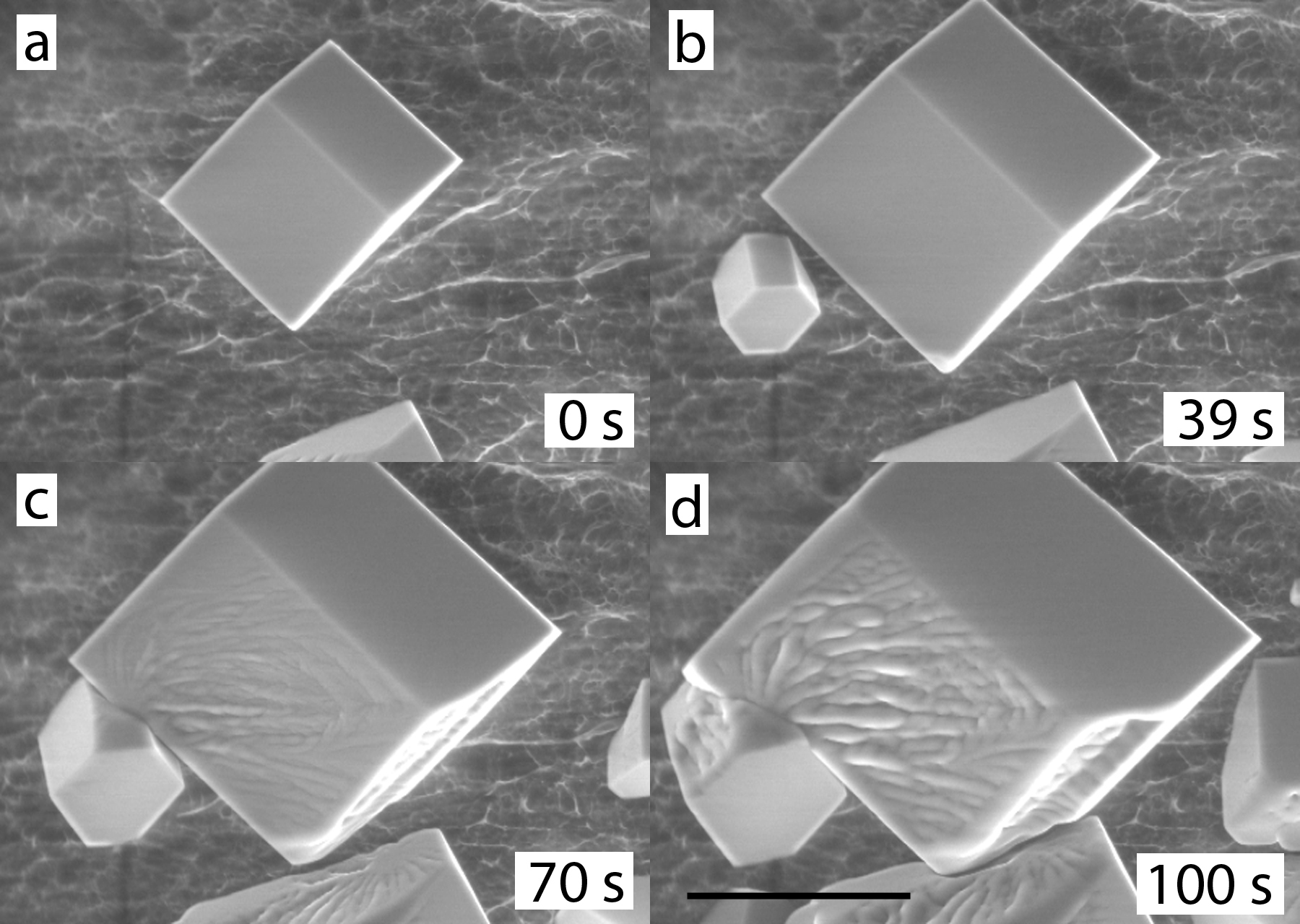

Supplement: Figure S2 — An example of grain boundary-induced surface transition, observed at a sample stage temperature of −15°C. The crystal surface temperature is estimated to between −13.6°C and −14.5°C. The water vapor density is 1.6 Torr. The scale bar in (d) is 100 µm. (TIF) [file pone.0024373.s003.tif]

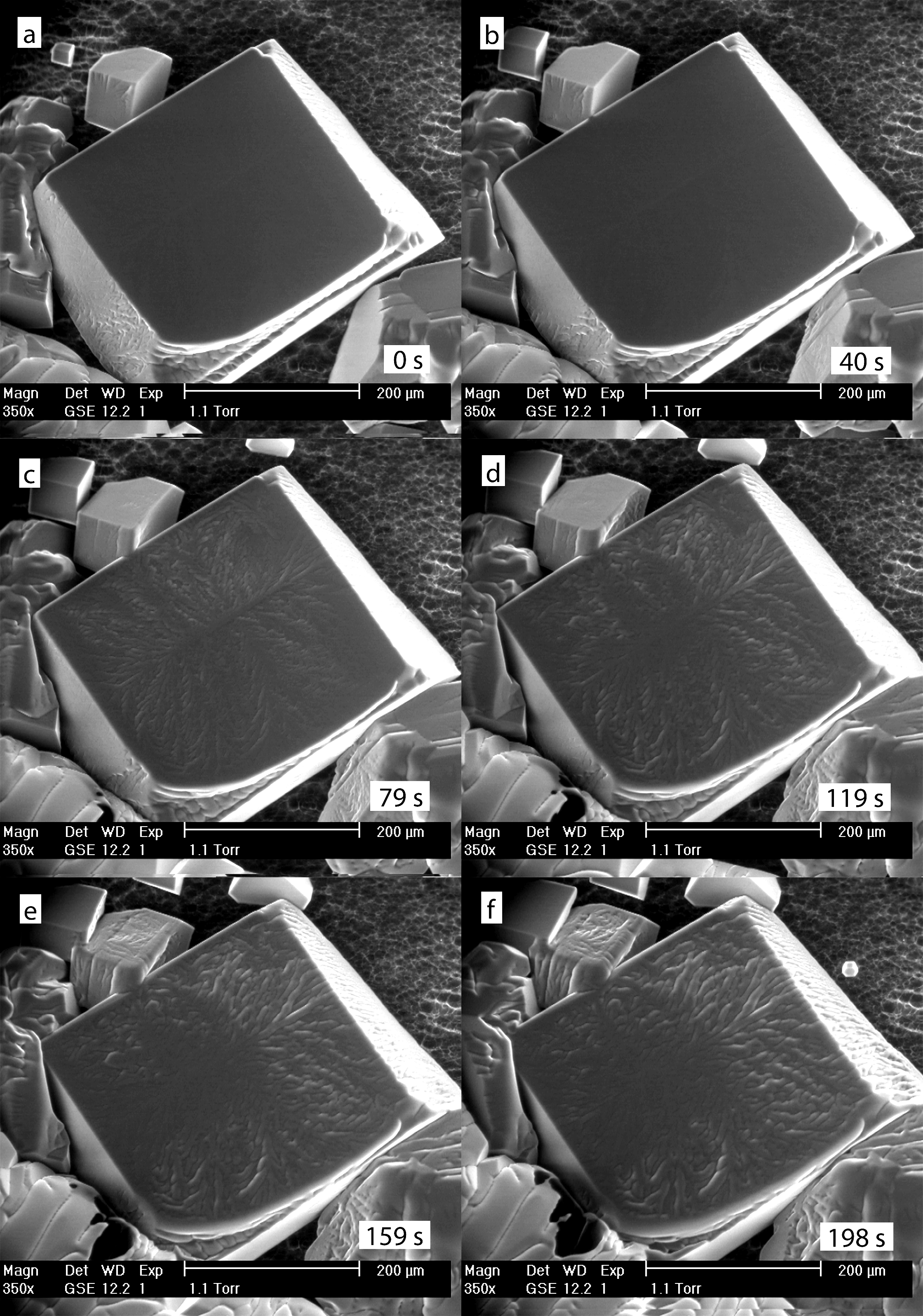

Supplement: Figure S3 — An example of a facet which grew to have edges longer than 200 µm before it came into contact with another crystal. In Figure S3b the facet appears perfectly smooth. In Figure S3c, the upper part of the facet has come into contact with another crystal, and the whole facet has undergone a surface transition. The sample stage temperature was −18.5°C, and the temperature of the crystal surface is estimated to between −17.3°C and −18.5°C. (TIF) [file pone.0024373.s004.tif]

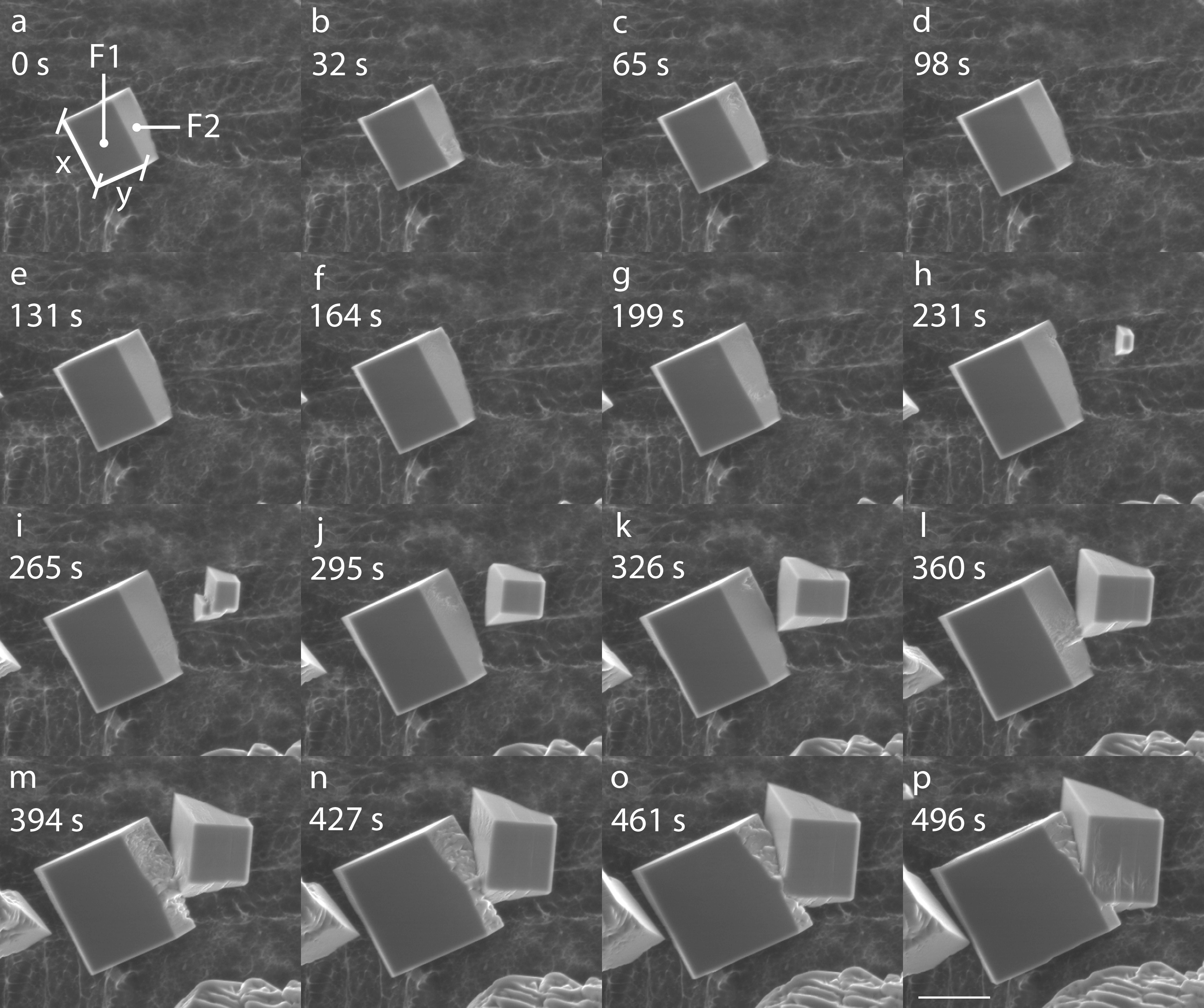

Supplement: Figure S4 — Increase in the linear growth rate of the facets which undergo grain boundary-induced surface transition. The figure shows two crystals that grow into contact, referred to as the left and the right crystal. For the left crystal, facets are named F1 and F2 while facet edges are named x and y, as shown in (a). Contact between the crystals is first observed in (l), and the contact results in a surface transition of facet F2. The x/y ratio of facet F1 is constant before the two crystals come into contact, but changes after contact is reached. The change in x/y ratio can be attributed to an increasing linear growth rate of facet F2. The water vapor density was 0.9 Torr. The temperature of the sample stage was −20°C, and the temperature of the crystal surface is estimated to between −18.4°C and −19.7°C. The scale bar in (p) is 50 µm. (TIF) [file pone.0024373.s005.tif]

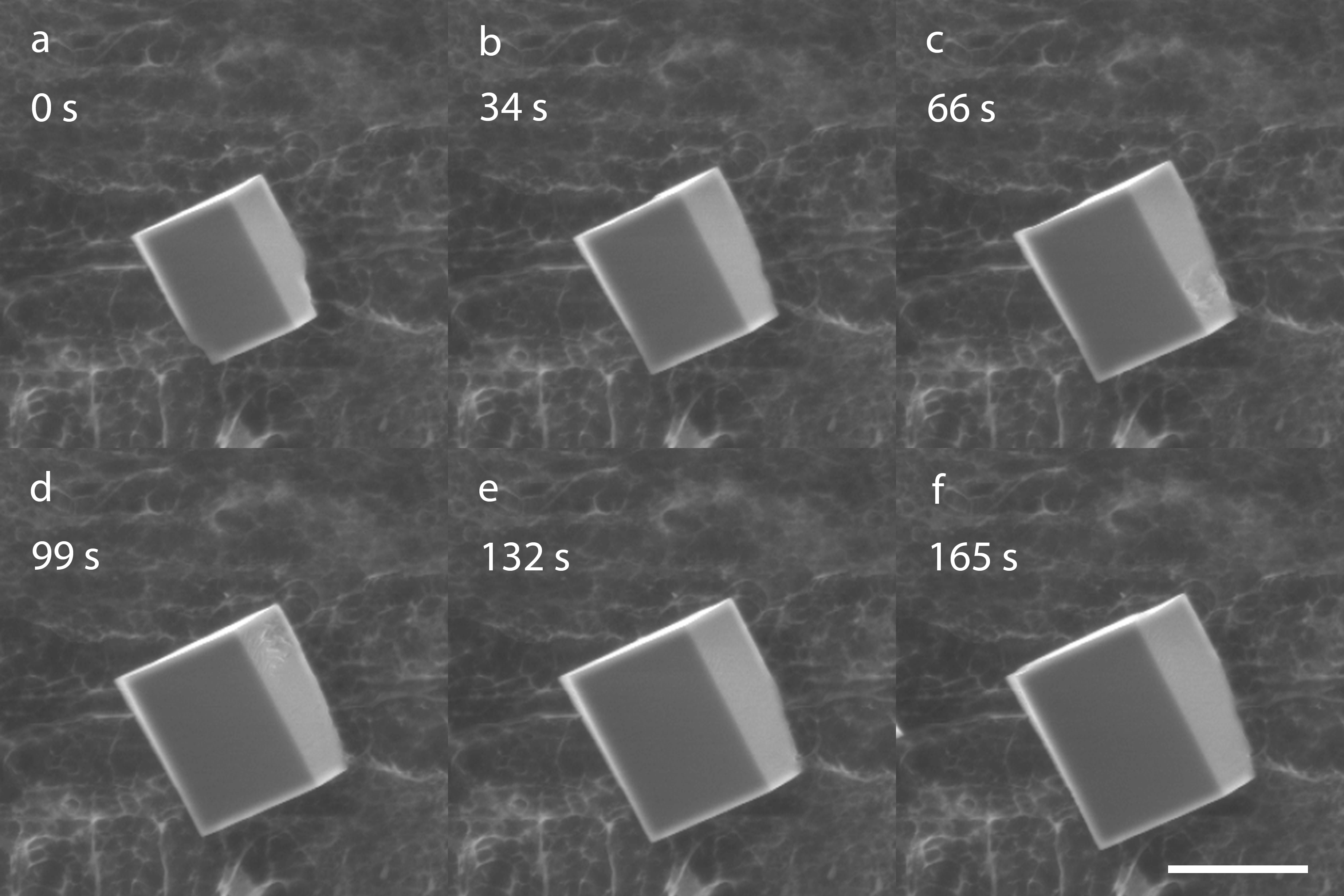

Supplement: Figure S5 — An example of a facet which develops an irregularity without contact with another crystal. Such irregularities generally disappeared relatively fast, as exemplified in this figure: the right facet appears perfectly smooth in (a–b), exhibits an irregularity in (c–d) and appears perfectly smooth in (e–f). The water vapor density was 0.9 Torr. The temperature of the sample stage was −20°C, and the temperature of the crystal surface is estimated to between −18.4°C and −19.7°C. The scale bar in (f) is 50 µm. (TIF) [file pone.0024373.s006.tif]
